# Market-based tools for accelerating cattle sustainability in Canada

**DOI:** 10.1093/af/vfab038

**Published:** 2021-09-06

**Authors:** Karen Haugen-Kozyra

**Affiliations:** Viresco Solutions Inc., Calgary, AB, Canada

**Keywords:** accelerating sustainability, Canadian beef production, low carbon beef, market-based tools, three-nitrooxypropanol

**Implications**

- Canada provides a useful and interesting model for tools that can accelerate livestock sustainability, in particular, for the cattle sector. Globally, cattle are responsible for about 65% of livestock’s greenhouse gas emissions, with methane from enteric fermentation of feedstuffs and manure accounting for about 44% of the emissions.
- This represents a unique opportunity for livestock-based solutions, facilitated by effective market mechanisms and tools, contributing to lower environmental impacts.
- This paper discusses key drivers enabling the opportunity and uses case studies from Canada that are operational today to demonstrate what could be possible in other jurisdictions given similar circumstances and capabilities.

## Introduction

As a country, Canada is an interesting microcosm of carbon pricing policies and sustainability tools. Carbon pricing is a tool that can accelerate decarbonization across sectors. Several provinces have had subnational carbon pricing schemes in place since the 2007 to 2008 timeframe (British Columbia, Quebec, and Alberta). Starting in 2016, the federal government under the Trudeau Administration worked with the provinces to establish the “Pan Canadian Framework”—a plan that strives to implement a pan-Canadian approach to pricing carbon pollution and measures to achieve reductions across all sectors of the economy. The road to harmonization has not been an easy or a popular one, with several provinces having since elected conservative governments currently challenging the constitutionality of the federal government’s imposition of backstop greenhouse gas regulations at the Supreme Court of Canada. Nevertheless, the carbon pricing systems remain in place, with the opportunity for the non-covered sectors such as agriculture, forestry, and waste treatment to voluntarily generate carbon offsets and sell to the regulated sectors for compliance-meeting purposes. It is this latter context—the ability to extend the price incentive to reduce the carbon intensity of agricultural production—that drove the development of tools to monetize activities for lower carbon beef production in Alberta, that is now extending to the Federal Offset System and beyond Canada’s borders. This paper will focus on the evolution of several market-based tools at play in Canada driven by subnational, national, sectoral certification systems, and global actions, policies, and pledges.

## The First Beef Carbon Credits in the World

As early as 2009, the Alberta Government approved three Carbon Offset Quantification Protocols incentivizing lower carbon beef production in the province—Feeding Edible Oils, Reducing greenhouse gas emissions (GHG) Intensity of Fed Cattle (Fed Cattle), and since 2012, Selection for Low Residual Feed Intake in Beef Cattle (Alberta Offset System 2021; https://www.alberta.ca/alberta-emission-offset-system.aspx). Alberta is the only jurisdiction in the world where project developers have been able to generate compliance-grade beef carbon offsets for sale to large final emitters in Alberta’s Carbon Pricing system. Trimble Corporation Canada has three projects listed on the Alberta Emissions Offset Registry under “The Reducing greenhouse gas Intensity of Fed Cattle Protocol” for practices implemented that increase feed efficiency of beef production. The projects generate over 49,000 tonnes of beef carbon offsets in four Alberta feedlots. In 2017, these tonnes were sold to a variety of buyers (Capital Power, Brightspot Climate, and TransAlta; https://alberta.csaregistries.ca/GHGR_Listing/AEOR_ListingDetail.aspx?ProjectId=229). Two more fed cattle projects are listed on the Registry by Trimble, generating carbon from 15 more feedlots in the 2017 project year, for an estimated total of over 90,000 tonnes of emission reductions altogether. Growsafe Beef Systems has one project listed for the “Selection for Low Residual Feed Intake in Beef Cattle protocol” on the Registry to date (https://www.csaregistries.ca/albertacarbonregistries/eor_project.cfm?id_prj=820). The Canadian government recognizes the quality of the Alberta cattle projects and announced that they would recognize these compliance units in the National Offset System. They have also signaled the development of livestock feed management protocols for the National Offset System as well, enabling projects in other provinces. Further, elements of Alberta’s cattle protocols can be found in Voluntary Carbon Offset Markets (Verra 2021; https://verra.org/methodology/methodology-reduce-enteric-methane-emissions-beef-cattle-using-organic-natural-feed-supplements and https://verra.org/methodology/reduction-of-enteric-methane-emissions/) and the Australian Emission Reduction Fund (https://www.industry.gov.au/regulations-and-standards/methods-for-the-emissions-reduction-fund). In 2018, the Alberta Dairy Protocol was adapted into the Gold Standards’ suite of offset methodologies by TREEs and DSM Nutritional Products (https://www.goldstandard.org/sites/default/files/documents/gs_agriculture_clean_cow_meth_dec_2018.pdf). In 2019, the Climate Action Reserve approved the first Canadian voluntary protocol dedicated to conserving grasslands in Canada and the carbon stored in the beef-pasture production system—Canadian grasslands represent one of the most intact ecosystems in the world and are critical for preserving biodiversity and contributing to climate mitigation. A pilot is underway to test the protocol across Canadian ranches.

## The First Jurisdictional-Certified Sustainable Beef Framework under the Global Roundtable

In 2011, McDonald’s was one of 12 founding members that initiated the Global Roundtable for Sustainable Beef (GRSB; https://grsbeef.org; original members included WWF, McDonald’s, Cargill, National Wildlife Federation, The Nature Conservancy, several other national affiliates, ranchers, and other stakeholders)—a multistakeholder, market transformation organization that brings together key players in the beef industry, from ranchers to retailers, helping to identify opportunities for continuous improvements in sustainability throughout the global beef supply chain. These efforts aim to minimize greenhouse gas emissions, protect native forests and grasslands with high conservation value, efficiently manage water use and quality, promote soil health, and protect biodiversity—all while maintaining the social and economic viability of beef production worldwide. These goals are outlined in the framework of the GRSB, and as part of the company’s commitment to sustainable beef, McDonald’s set a pioneering goal in early 2014 to begin sourcing sustainable beef by 2016. Since the goals, principles, and criteria of the GRSB had not yet been implemented at a local level, launching a Sustainable Beef Pilot was the next step in McDonald’s long-term strategy to achieve its 2016 goal, and they chose Canada as the Pilot country. The reasons McDonald’s chose Canada to pilot the translation of the GRSB goals, principles, and criteria were because Canada had a national traceability system, a consolidated supply chain with the Cargill patty plant in Alberta supplying the Country’s restaurants, a supportive set of industry programs to base the Pilot on, and available funding for implementation (note: in more complex and disaggregated supply chains distributed across more diverse geographic regions, the approach may have to be more customized to localized supply chains). Beginning in 2015, the Pilot set out to develop indicators of sustainable beef production that were outcome-based, in keeping with the GRSB’s intended means of verification (as described in the GRSB’s “Principles and Criteria for Sustainable Beef” document; https://grsbeef.org/WhatIsSustainableBeef). Outcome-based metrics allow each producer to describe how they deliver the positive outcomes associated with a given indicator, rather than requiring the producer to stick to a prescribed list of practices. The way cattle are raised in Canada varies across landscapes, enterprises, and production stages, so basing performance on outcomes enables different production systems to achieve the same objectives without mandating exactly how they get there. An outcome-based approach also protects the autonomy of individual producers to make decisions that best suit their own unique resources and business interests. This effort required enlisting the help of 11 respected advisors (i.e., the advisors were selected from the general principle categories outlined in the GRSB framework; http://www.mcdvsb.com) and gathering insights from dozens of discussions with Canadian ranchers, feedlot operators, and processors as well as representatives from retail, foodservice, academia, nongovernmental organizations, government, and industry associations. Once the indicators for cow–calf, backgrounding, and feedlot operations were drafted and McDonald’s had confirmed the decision to verify based on outcomes, the Pilot team needed to establish a consistent method of scoring individual performance across the indicator and operation sets. A performance scale was developed and tested through significant feedback from advisors and other Canadian beef industry subject matter experts. The testing involved experienced, independent third-party verifiers (http://wherefoodcomesfrom.com) to assign and calibrate with each other a sample of operations with performance scores for each indicator using the three techniques of interview, observation, and records-checking during verification. The Pilot ended in mid-2016 proving that beef from sustainable sources could be successfully tracked through the Beef Information Exchange (BIX; https://www.trustbix.com). This created a beef sustainability system that verified Canadian operations as sustainable and then tracked cattle chain of custody through these operations into the two processors that supply McDonald’s with their beef in Canada. This translated to nearly 8 million lbs of Canadian hot carcass weight delivered by an entirely verified sustainable supply chain. Using a mass balance calculation, McDonald’s sourced just over 300,000 lbs of Canadian beef trim from entirely sustainable sources during the Pilot (http://www.mcdvsb.com).

At the beginning of 2018, the Canadian Roundtable on Sustainable Beef (CRSB) launched the Certified Sustainable Beef Framework through a multistakeholder development process, including two rounds of public consultation (https://www.crsbcertifiedsustainablebeef.ca; the CRSB relied on the guidelines and communications with experts in the International Social and Environmental Accreditation and Labeling (ISEAL) Alliance to help develop the certification framework). This innovative effort is essentially the first of its kind for the beef industry globally, aligned with the Five Principles of Sustainable Beef as set by the GRSB. There are similar programs in other commodities, such as seafood, forest and paper products, sugar, coffee, palm oil, and others upon which the CRSB has drawn from lessons learned and in accordance with ISEAL Alliance guidelines (https://www.isealalliance.org/defining-credible-practice). Most of these programs are internationally based. There are similar organizations and Roundtables for Sustainable Beef in other countries, such as the United States, Brazil, Australia, Columbia, and others; however, Canada is the first to build such a robust certification framework with chain of custody requirements and claims to support its Standards. Other countries are following the leadership position that the CRSB is taking with keen interest.

**Figure 1.**
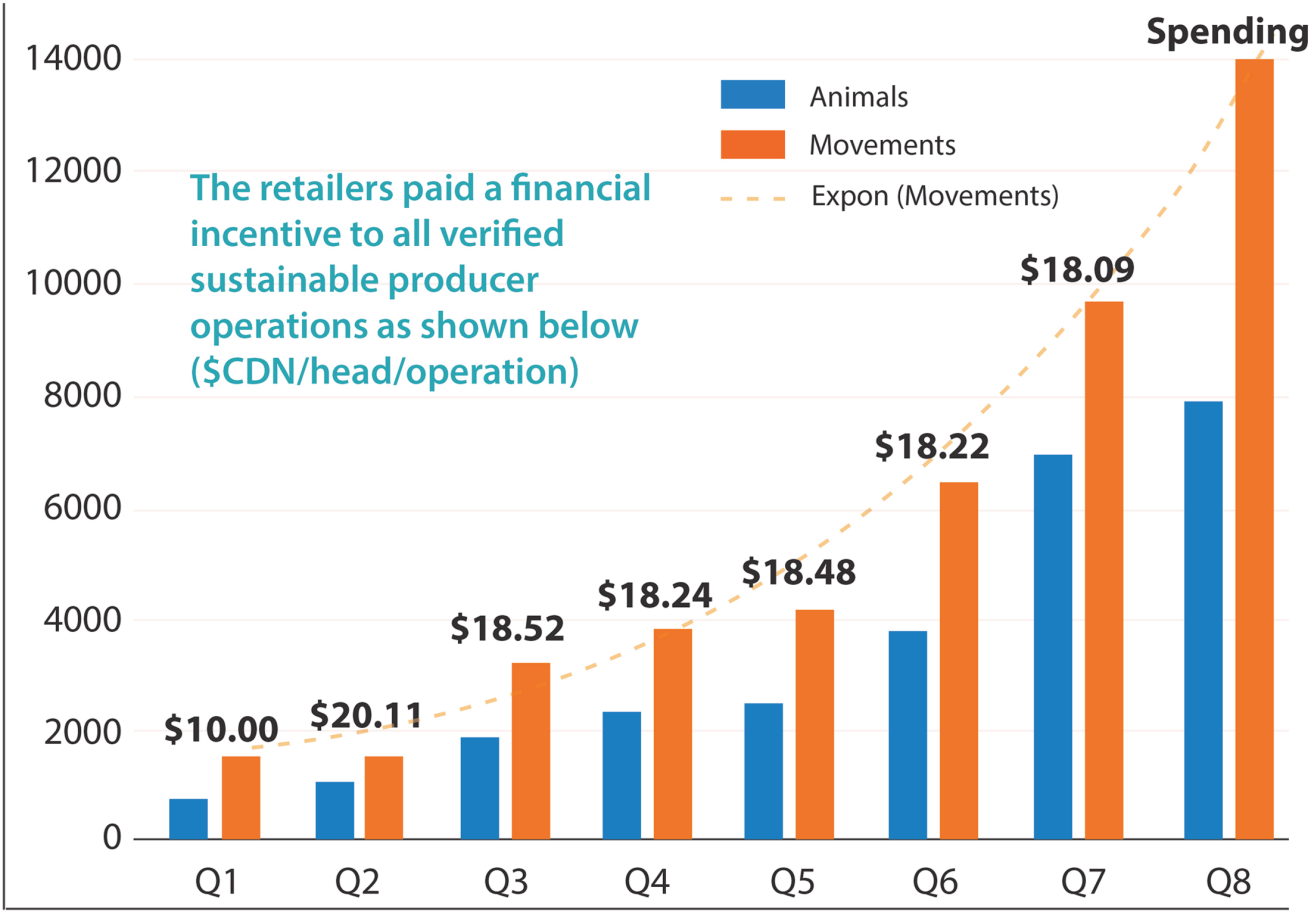
Uptake in the CBSA Pilot shows a 25% increase in the qualified volume of certified beef every quarter, along with increased value per head per operation ($CDN/head/operation).

To further accelerate the certification of sustainable beef operations in Canada, and grow the traceable supply of certified sustainable beef, Cargill and partners TrustBIX (BIX) and VBP+ (Verified Beef Production plus) launched an innovative Certified Beef Sustainability Acceleration (CBSA) pilot that ran from 2017 to 2019 (https://cbsapilot.ca; the Pilot is now a Program with long-term commercial stability providing enhanced economic viability to beef operations in Canada and ensuring the sustainability of Canada’s beef value chain). A growing number of retailers joined the CBSA pilot evolving it into a longer-term program—McDonald’s, Loblaws, Original Joe’s, Swiss Chalet, Cactus Club Café, Harvey’s, and Centennial Foods, and the list is growing. Collectively, the retailers pay premiums back to beef producers for fully certified sustainable beef tracked through the TrustBIX platform. BIXS tracks all of the cattle on an radio frequency identification (RFID) tag basis passing through operations that are certified and, then, using distributed ledger technology, issues cheques to the beef operators on behalf of retailers. The premium-enhanced uptake tracked by BIXS’ innovative technology-based platform, coupled with the Certified Sustainable Beef programming, initially catalyzed by McDonald’s Sustainable Beef Pilot, demonstrates what is possible through market-based tools and how others could do it through meaningful partnerships—it’s not Just a Flash in the Pan (not just a flash in the pan: https://www.youtube.com/watch?v=QJV6-Y2rCYc&feature=youtu.be; Figure 1).

By the time the Pilot ended in September 2019, close to Canadian $1.8 million was paid to beef producers participating in the Pilot. Now that it has become a program, with more retailers coming on board, the success story will grow even larger.

## Expanding Market-Based Tools for Sustainable Cattle Production

A number of global drivers are catalyzing the development of low-carbon cattle production. Finding ways to grow more food without growing greenhouse gas emissions is the mandate of the Global Research Alliance (GRA). After all, greenhouse gas emissions represent a loss of costly inputs—both nutrients and feed energy—ultimately resulting in inefficiencies in agricultural production systems. Cattle (raised for both beef and milk) are responsible for about 65% of the livestock sector’s emissions, with methane from enteric fermentation of feedstuffs and manure accounting for about 44% of the emissions (Gerber et al., 2013).

**Figure 2.**
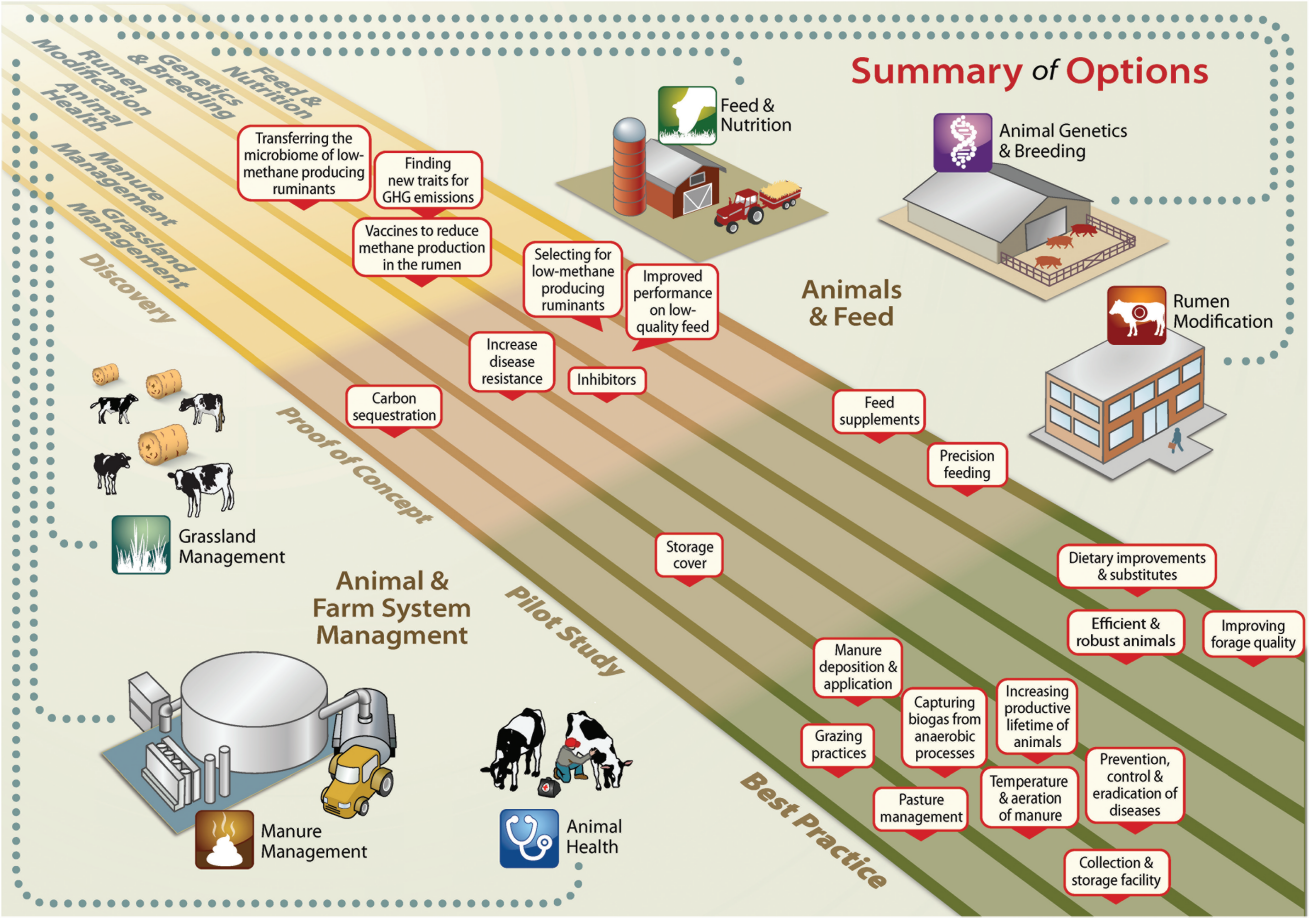
Adapted from the LRG’s Best Practice and Emerging Options report. Each option is categorized by whether they are best practices, candidates for pilots, at a proof of concept stage or the discovery stage requiring more applied research (http://www.saiplatform.org/uploads/Modules/Library/lrg-sai-livestock-mitigation_web2.pdf).

The Livestock Research Group (LRG) of the GRA, chaired by New Zealand, Ireland, and the UK, focuses on actions to reduce the emissions intensity of livestock while increasing food security. In fall 2016, the LRG and the Sustainable Agriculture Index (SAI) Platform released a report summarizing current climate smart practices implementable today and in the near term, and where industry partners, farm group agencies, and policy groups could collaborate further in the development, trial, and deployment of more mitigation options. The SAI Platform president points out that although solutions can be found in science, if ranchers/farmers do not find them relevant, useful, or actionable—they won’t go anywhere. The report charts a useful roadmap with the main options for reducing greenhouse gas emissions, improving productivity, and enhancing food security and animal health are: 1) animal breeding/genetics, 2) rumen modification, 3) grassland management, 4) manure management, and 5) animal health (Figure 2).

**Figure 3.**
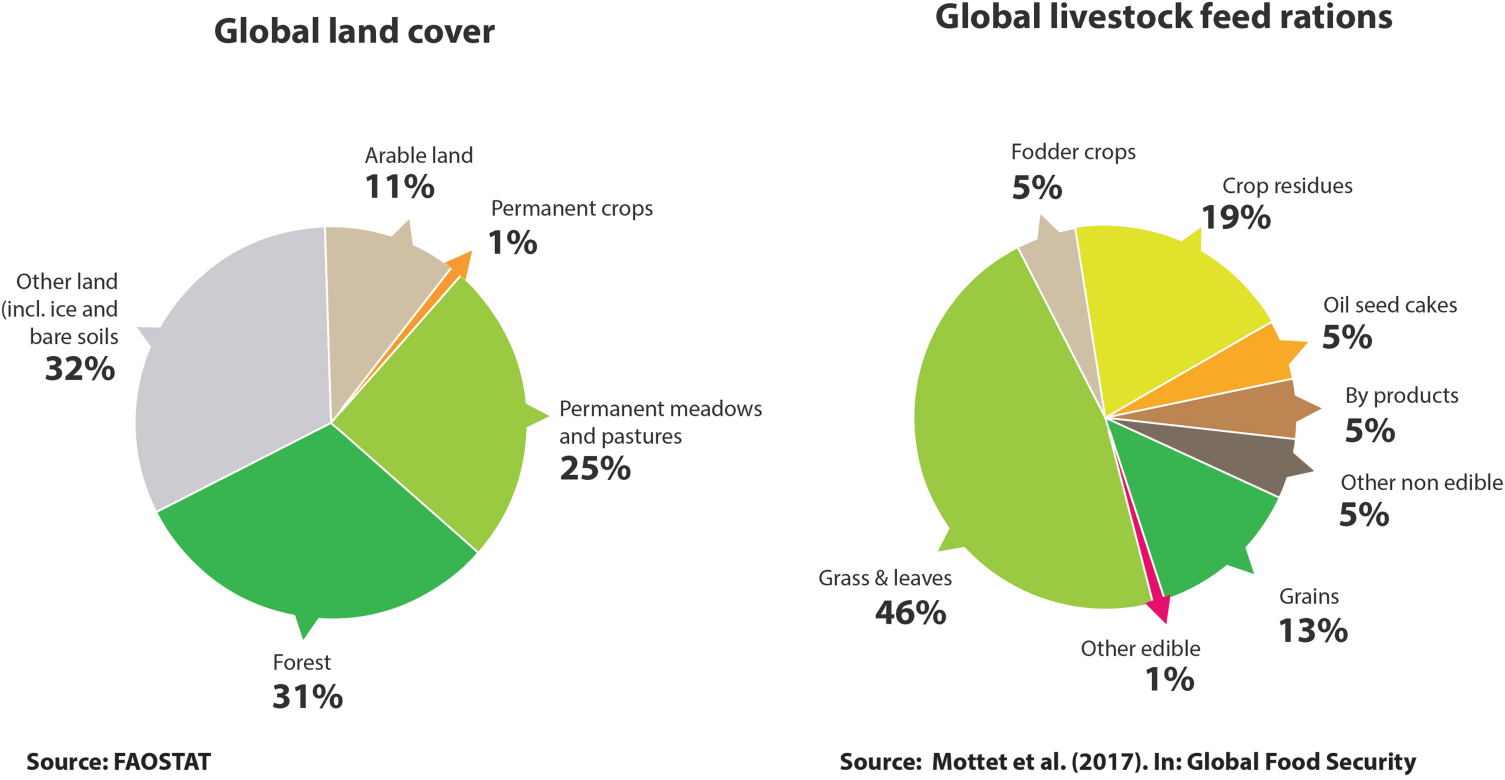
Global land cover classes and livestock feed rations (courtesy of FAO, 2017).

Similarly, the Food and Agriculture Organization (FAO) of the UN published a Brief in 2017 outlining three major livestock solutions for climate change (FAO, 2017): 1) productivity improvements to reduce livestock carbon intensity, 2) carbon sequestration, and 3) better livestock integration into the circular bioeconomy (http://www.fao.org/3/a-i8098e.pdf). In the Brief, the FAO authors point out that although grasslands represent less of the global landcover than forests, they are estimated to contain 343 billion tonnes of carbon—nearly 50% more than is stored in forests worldwide and are at less risk from catastrophic events like fire (Figure 3).

In Canada, the production efficiency of the beef sector has increased steadily since at least 1981. Life cycle GHG assessments of Canadian beef production have reported that in 2011, the same amount of beef required 29% less breeding stock, 27% fewer slaughter cattle, 24% less land, and produced 15% fewer GHGs than in 1981 (McAllister et al., 2015; Legesse et al., 2016). The cow–calf sector specifically gained efficiency through improved nutrition, growth implants, health, crossbreeding, and pasture management. Although many think the low-hanging fruit opportunities have been captured, there still exist opportunities for accelerating low-carbon beef production.

In a recent study prepared for the Alberta Beef Producers, Viresco Solutions categorized available and near-term best management practices (BMPs) with the potential to reduce GHG emissions according to science-based investigations. The BMPs were also classified according to modes of action, where applicable:

1. Reduced GHG intensity (CO_2_ equivalent per kilogram of beef)
  a) Management practices that improve production efficiency (e.g., reducing age at slaughter, fewer days in the feedlot, beta-agonists, growth implants, and culling open cows).
  b) Animal Breeding—crossbreeding for optimum hybrid vigor, which improves female fertility, longevity, and lifetime productivity (maternal heterosis) and possibly calf health (heterosis of the individual).
  c) Animal Genetics—selecting for low residual feed intake (RFI), which reduces methane emissions (g/d) and methane yield (g/kg dry matter intake [DMI]).
  d) Winter grazing that reduces CO_2_ emissions from burning fossil fuels to distribute feed and heat winter housing.
  e) Manure management practices that reduce methane emissions from excreta, including the circular bioeconomy (e.g., Biogas)
2. Rumen modification
  a) Methanogenesis inhibitors (e.g., three-nitrooxypropanol [3-NOP], *Asparagopsis*, and lemongrass).
  b) Plant compounds (e.g., tannins).
  c) Edible oils and fats.
  d) Possibly biochar—ongoing research into the use of biochar as a methanogen inhibitor indicates this could be a future BMP.
3. Feed and nutrition
  a) Improving diet quality
  b) Feed supplementation
4. Grassland management and retention
  a) Soil carbon sequestration
  b) Avoiding loss of soil organic carbon stores

Generally, overall efficiency can be increased through a variety of practices that, through their combined impacts, are almost guaranteed to result in reduced production costs as well as reduced carbon intensity. In a 2017 analysis of Canadian beef production, many management practices and indicators separated “low-emitting” (mean = 19.9 kg CO_2_e/kg live weight sold, SD = 1.23) from “high-emitting” (mean = 28.7 Kg CO_2_e/kg live weight sold, SD = 2.55) farms. These practices led to a 31% reduction in average emission intensity for low-emitting farms (Alemu et al., 2017a and 2017b). These indicators included:

- Lower retention of less efficient cows
- Clear culling (replacement) criteria—reproductive efficiency
- Early calving season
- Higher calf birth weight—although concerns are raised over calving difficulty/dystocia (Holland and Odde, 1992)
- Higher calf weaning weight
- Increased total live weight sold
- Higher beef productivity (live weight sold per unit ha, in the case of extensive cattle production systems)
- Improved productivity per animal (live weight sold/Animal Unit)
- Minimal purchased cereal grain and forage per unit cow
- Higher total digestible nutrient of feedstuffs
- Protein levels balanced to meet requirements
- Reduced land used for annual crop forage
- Increased land for perennial forage and pasture
- Reduced N fertilizer application on forage and pasture (incorporate legume and recycle manure)
- Increased proportion of stockpiled manure.

Additionally, more efficient and faster-growing cattle will reach slaughter weight faster and emit less greenhouse gases over a shorter time period. Although fewer days on the feed will inherently result in lower greenhouse gas emissions in feedlot systems, White & Capper (2013) compared the effect of focusing on average daily gain (ADG) and finishing weight as a metric of robustness in cow–calf systems. The study found that the producers who focused on increasing finishing weight by 15% performed better than those who focused on increasing ADG by 15% through better feed consumption, water use, and land use benefits. Increased ADG resulted in a 51% to 117% increase in sector profitability for cow–calf producers, while finishing weight change in cow–calf profit ranged from 67% to 143%.

Cattle that use less feed per pound of gained weight than other cattle are known as having a low/negative RFI or a negative net feed efficiency resulting in fewer greenhouse gas emissions from enteric fermentation and manure (Alberta Agriculture and Food, 2007). These cattle can be selected and tested for an RFI value or may be selected for on-farm breeding due to their observed feed utilization efficiency. Estimates report up to a 28% reduction in GHGs for low RFI cattle, on average (Asgedom and Kebreab, 2011). Low RFI cattle produce 15% to 25% less enteric methane (19.82 kg CO_2_e/kg carcass beef for low RFI vs. 23.06 kg CO_2_e/kg carcass beef), with lower DMI and improved feed conversion ratio, a 1% to 2% improvement in dry matter and crude protein digestibility, and 13% less farm area (Basarab et al., 2013).

The UN FAO identified RFI as having the greatest potential in Canada, as a high input country, through genetic/genomic selection for fertility (FAO, 2013). New tools are being generated to aid in the ease of implementing genetic efficiency programs, such as the Envigour HX genomic tool. The tool combines parentage verification, genomic breed composition, and an assessment of hybrid vigor to generate a genomically determined hybrid vigor score. Newly published research reveals that a high vigor cow herd, as determined from genomic breed composition and retained heterozygosity, has improved fertility, longevity, and lifetime productivity compared with a low vigor herd (Basarab et al., 2018) and a significantly lowered carbon footprint (the genetic tools presented here have not been found to affect undesirable recessive traits within the population to date).

Several potential methane inhibitors are under development, with the most promising and researched inhibitor called three-nitrooxypropanol (3-NOP), from Royal DSM based in the Netherlands; 3-NOP is a feed-based additive that destabilizes the enzyme in the last step of the methanogenesis pathway in the rumen bacteria *Archea* sp. Global independent studies have shown emission reductions of at least 30% in research studies in sheep, beef, and dairy cattle. Researchers at Agriculture and Agri-Food Canada have been researching this feed additive for the last 5 years with good results on both GHG reductions and performance improvements, in typical backgrounding and finishing diets in Alberta (Romero-Perez et al., 2014, 2015; Vyas et al., 2016a, 2016b). Viresco Solutions, along with partners Royal DSM, Ag Canada, and Feedlot Health, coordinated the largest 3-NOP demonstration trial in the world (https://eralberta.ca/projects/details/demonstration-reduced-enteric-methane-emissions-growingfinishing-beef-cattle/). Emissions Reduction Alberta committed $1.5 million to this $3 million project through its *Methane Challenge*. The project was recognized for having positive implications for the province due to the fact that 70% of Canada’s cattle production happens in Alberta. With ~15,000 cattle included in the trial, it represents the largest single trial conducted on methane reduction technologies for ruminants. Measurements indicated that an average 70% enteric methane emission reduction was found when the feed ingredient was provided in steam-flaked or dry-rolled barley finishing diets. In steam-flaked corn, a reduction in the range of 31% to 80% was observed. In backgrounding diets, increasing the dose of the feed ingredient stepwise decreased the yield of methane by 17% to 26% compared with control animals. The trial successfully demonstrated that the ingredient can be included in commercial feedlot diets to reduce methane emissions, without negative effects on animal health and performance parameters and carcass characteristics (McGinn et al., 2019;Alemu et al., 2021a, 2021b).

**Figure 4.**
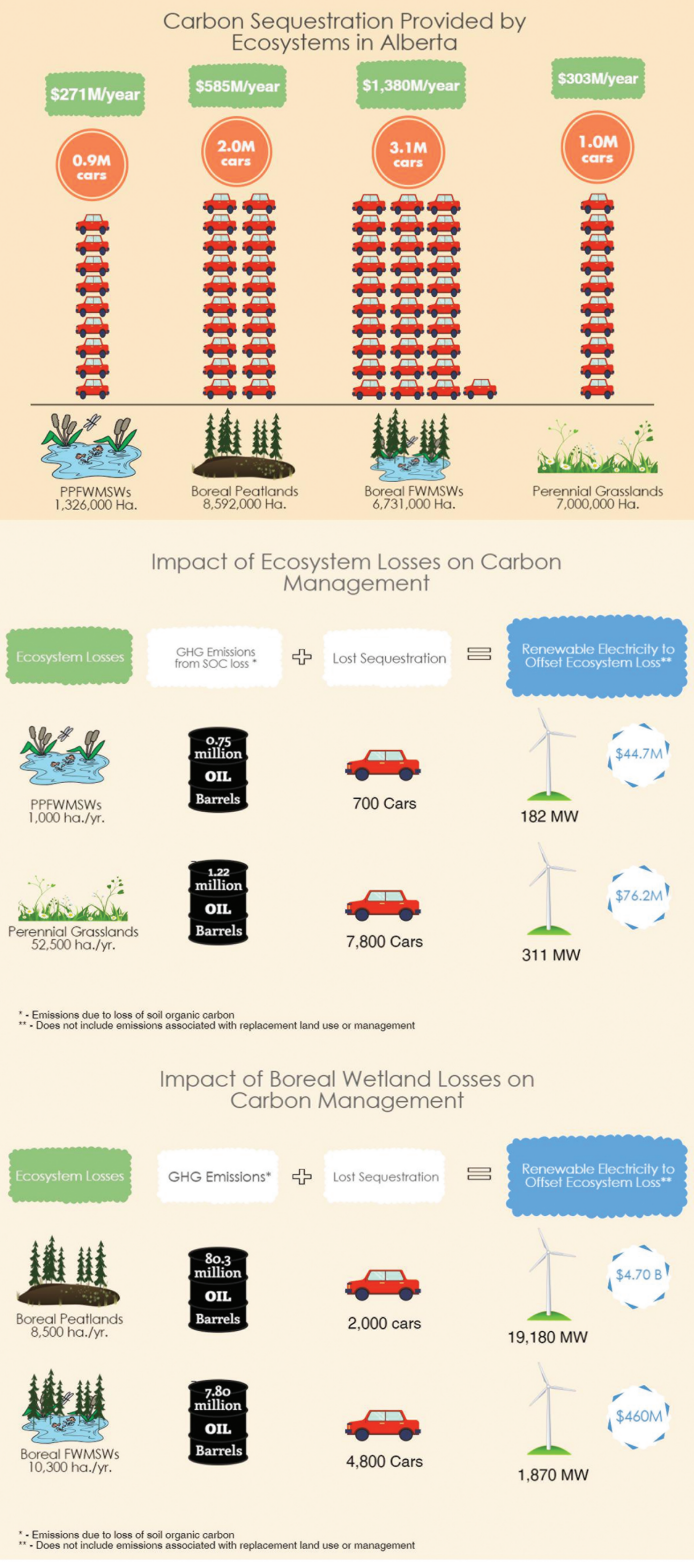
The value of all perennial grasslands (far right) as well as wetlands (far left) in the white zone, Boreal ecosystems in the green zone of Alberta (Viresco et al., 2017; https://virescosolutions.com/wetlandsgrasslands-initiative/), and soil organic carbon losses from ongoing annual ecosystem losses in Alberta with average carbon value applied to estimated carbon pricess to 2030 (Viresco Solutions Inc., 2017).

In an analysis of Natural Climate Solutions (NCS) pathways for Canada, Nature United (The Nature Conservancy in Canada) assessed readily deployable options that can contribute to Canada’s goals for emissions reductions—from the protection, management, and restoration of natural systems (Drever et al., 2021). The study estimated NCS pathways can provide up to 78.2 (41.0 to 115.1) Tg CO_2_e (95% confidence interval [CI]) of mitigation annually in 2030, and 392.8 (173.2 to 612.4) Tg CO_2_e between 2021 and 2030, with 34% available at ≤ CAD $50/Mg CO_2_e. Among the largest mitigation opportunities is avoided conversion of grasslands (retaining grasslands through market mechanisms). In a recent study conducted by Viresco, the contribution of existing grasslands/pasture carbon sequestration in Alberta was equivalent to $303M/yr ($43/ha at predicted carbon prices from $30/tCO_2_e 2018 to $100/tCO_2_e in 2030 (Sawyer and Bataille, 2017)) or taking nearly 1 M cars off the road every year (Figure 4). Retention of Prairie Pothole Freshwater Mineral Soil Wetlands that are often co-located with grasslands also adds significant carbon sequestration capacity.

Further, annual losses of soil organic carbon were extracted from current estimated rates of ecosystem losses in Alberta and applied to predicted carbon prices to 2030 from the study of Sawyer and Bataille (2017) and Viresco Solutions Inc. (2017) (Figure 4).

As mentioned previously, a Pilot is underway in Canada to pressure test the newly approved Canadian Grassland Project Protocol under the voluntary Climate Action Reserve Registry and Standard. This will be an important avenue to realize the carbon and other co-benefits from preserving Canadian grasslands, and response by Canadian ranchers to participate has been strong and buyers of these carbon offsets have shown interest in carbon and other outcomes from the preservation of grasslands.

## Conclusions

Taken together, all of the activities mentioned in this report, and outlined in Table 1, represent a possible 50% reduction of greenhouse gases from cattle operations in Canada in the next few years given the right market incentives (using 3-NOP and other feed additives, winter grazing, genomic hybrid vigor, reduced age at slaughter, good management practices, and diet formulation). In conjunction with soil organic carbon retained under Canada’s grasslands and pastures with market-based mechanisms like the Avoided Conversion of Grasslands protocol, Carbon Neutral Beef production in Canada is feasible.

**Table 1.** Summary of selected BMPs

BMP	Category	Mode of action	Current status in Canada
Green practices			
Maintain efficient, robust, and fertile animals	Livestock management	1(a,b)—Reduced GHG intensity	Common practice
Use growth promotants	Livestock management	1(a,b)—Reduced GHG Intensity	Common practice
Implement calf-fed timelines and culling programs	Livestock management	1(a,b)—Reduced GHG Intensity	Readily available, with variable implementation
Select for low-methane-producing livestock	Livestock management	1(c)—Reduced GHG Intensity	Readily available, with limited implementation
Extend the grazing season and overwintering	Soil and land management	1(d)—Reduced GHG intensity & 4(a)—Grassland Management and Retention	Readily available, with variable implementation
Manure nutrient management	Manure management	1(e)—Reduced GHG Intensity	Common practice
Dietary improvement and substitutes	Feed management	3(a)—Feed and Nutrition	Common practice
Improve forage quality	Feed management	3(a)—Feed and Nutrition	Common practice
Improve grazing for soil health	Soil and land management	4(a)—Grassland Management and Retention	Readily available, with variable implementation
Improve pasture management	Soil and land management	4(a)—Grassland Management and Retention	Readily available, with variable implementation
Convert marginal cropland to perennials	Crop management	4(a)—Grassland Management and Retention	Readily available, with variable implementation
Reseed forages	Crop management	4(a)—Grassland Management and Retention	Readily available, with variable implementation
Incorporate legumes and pulses	Crop management	4(a)—Grassland Management and Retention	Readily available, with variable implementation
Orange practices			
Anaerobic digesters	Manure management	1(e)—Reduced GHG intensity	Technology is available, with limited implementation
Feed supplements	Feed management	2(b,c,d)—Rumen Modification & 3(b)—Feed and Nutrition	Variable availability and implementation, still being developed in several cases
Blue practices			
Precision ranching	Livestock management	1(a)—Reduced GHG intensity	Being piloted—technology is still being developed
Improved performance on low-quality feed	Livestock management	2(a)—Rumen modification	Proof of concept
Methane inhibitors	Livestock management	2(a)—Rumen modification	Demonstration Stage; regulatory approvals needed
Transferring the microbiome of low-methane-producing ruminants	Livestock management	2(a)—Rumen modification	Discovery stage
Vaccines to reduce methane production in the rumen	Livestock management	2(a)—Rumen modification	Discovery stage
Precision feed tracking	Feed management	3(a)—Feed and Nutrition	Being piloted—technology is still being developed
Management of riparian areas	Soil and land management	4(a)—Grassland management and retention	Practices have been tested but are limited by small area
Silvopasture	Crop management	4(a)—Grassland management and retention	Being piloted

Organized by color category followed by mode of action: Green—good reduction potential, ready to implement; Orange—has tradeoffs and currently is not practical to implement; Blue—Under development and still within proof of concept/demonstration stage.

## About the Author

**Karen Haugen-Kozyra** is the President of Viresco Solutions Inc. (www.virescosolutions.com), a network of leading advisors helping agriculture, bioenergy, and sustainable food sectors navigate the nexus between lowering their carbon footprint and developing an overall sustainability strategy. She has over 30 years of experience in agricultural GHG measurement/modeling and environmental policy development from the private sector and at the provincial Department of Agriculture, Climate Change Central. She has measured soil organic carbon (C) change and GHG in a network of sustainable cropping system sites across Alberta, developed the infrastructure and protocols necessary to support compliance level C-credit trading in Alberta, and adapted 13 C-offset protocols into the California, Quebec, and Ontario linked C-markets under the Western Climate Initiative. Viresco is working with partners to pilot a C-accounting and insetting framework for cost-effective reductions at scale from no-till and cover cropping (the second certification for an Ag Project under SustainCert) and testing the new module of the Cool Farm Tool to beef production systems with the Alberta Beef Producers.
